# Wide-Temperature Tunable Phonon Thermal Switch Based on Ferroelectric Domain Walls of Tetragonal KTN Single Crystal

**DOI:** 10.3390/nano13030376

**Published:** 2023-01-17

**Authors:** Shaodong Zhang, Shuangru Li, Lei Wei, Huadi Zhang, Xuping Wang, Bing Liu, Yuanyuan Zhang, Rui Zhang, Chengcheng Qiu

**Affiliations:** 1 Advanced Materials Institute, Qilu University of Technology (Shandong Academy of Sciences), Jinan 250014, China; 2 Shandong Academy of Sciences Yida Technology Consulting Co., Ltd., Jinan 250014, China; 3 Key Laboratory for Light Conversion Materials and Technology of Shandong Academy of Sciences, Advanced Materials Institute, Qilu University of Technology (Shandong Academy of Sciences), Jinan 250014, China

**Keywords:** ferroelectric domain walls, thermal conductivity, thermal switch, molecular dynamics, thermal boundary resistance

## Abstract

Ferroelectric domain walls (DWs) of perovskite oxide materials, which can be written and erased by an external electric field, offer the possibility to dynamically manipulate phonon scattering and thermal flux behavior. Different from previous ferroelectric materials, such as BaTiO_3_, PbTiO_3_, etc., with an immutable and low Curie temperature. The Curie temperature of perovskite oxide KTa_1−x_Nb_x_O_3_ (KTN) crystal can be tuned by altering the Ta/Nb ratio. In this work, the ferroelectric KTa_0.6_Nb_0.4_O_3_ (KTN) single crystal is obtained by the Czochralski method. To understand the role of ferroelectric domains in thermal transport behavior, we perform a nonequilibrium molecular dynamics (NEMD) calculation on monodomain and 90° DWs of KTN at room temperature. The calculated thermal conductivity of monodomain KTN is 9.84 W/(m·k), consistent with experimental results of 8.96 W/(m·k), and distinctly decreased with the number of DWs indicating the outstanding performance of the thermal switch. We further evaluate the thermal boundary resistance (TBR) of KTN DWs. An interfacial thermal resistance value of 2.29 × 10^−9^ K·m^2^/W and a large thermal switch ratio of 4.76 was obtained for a single DW of KTN. Our study shows that the ferroelectric KTN can provide great potential for the application of thermal switch at room temperature and over a broad temperature range.

## 1. Introduction

Thermal conductivity is of crucial importance for the application of materials in the areas of heat dissipation of microelectronics and chips, thermoelectric refrigeration, and thermal barrier coating [1]. In recent years, thermal runaway and the low-temperature effect caused by temperature changes can degrade chip performance and even cause safety hazards [2]. The high demands of the thermal management field require thermal functional materials possessing not merely high or low values of thermal conductivity, but also the ability to flexibly manipulate thermal fluxes by ambient temperature. However, controlling heat conduction has always been a challenging task. As the dominant heat carrier in insulating or semiconducting materials, phonons cannot be directly controlled by an external electric or magnetic field since phonons have no mass and no bare charge [3]. Previous efforts have been made by introducing particle or wave interference, e.g., structural inhomogeneities or periodic superlattices, to tune the propagation of phonons [4,5,6]. However, none of the techniques can provide a swift solution to dynamically tuning thermal transport over a broad temperature range.

Perovskite ferroelectric single-crystal materials offer the possibility to solve these problems. The peculiar ferroelectric domains of ferroelectric crystals, being aroused by spontaneous polarization when the temperature drops down to the Curie point, possess inherent electric dipole moments. The domain walls (DWs), a special nanoscale interface that separates uniformly polarized domains, can be generated or disappeared by an external electric field and phonon transport behavior, as well as thermal flux, which can be flexibly switched on and off accordingly [7,8,9]. Mante [10] and Weilert [11] et al., experimentally tuned the thermal conductivity of ferroelectric BaTiO_3_ and KH_2_PO_4_ by altering the density of DW under an electric field [12]. Theoretical work was carried out by Juan and Royo et al. [13,14] to evaluate the thermal boundary resistance (TBR) and interface phonon transport of ferroelectric PbTiO_3_ DWs within nonequilibrium molecular dynamics (NEMD) and nonequilibrium Green’s function (NEGF) approaches. Langenberg and Hopkins et al. carried out the study of TBR by experimental means in recent years [15,16]. However, the Curie temperature of the above-mentioned ferroelectric materials is very low and fixed, which apparently hinders the thermal switch operation at a broad temperature range, especially at room temperature.

In this work, the ferroelectric material KTa_1−x_Nb_x_O_3_ (KTN) is studied in order to solve this problem. As the solid solution of KTaO_3_ (KT) and KNbO_3_ (KN), the Curie temperature of KTN crystal can be regulated by changing the ratio of Ta/Nb. To obtain the ferroelectric phase at room temperature, a tetragonal KTa_0.6_Nb_0.4_O_3_ (KTN) single crystal is grown by the Czochralski method [17]. We performed a NEMD simulation to study the thermal transport behavior of KTN in the mono- and multidomain (90° DWs) ferroelectric states at room temperature. Combined with the experimental results of tetragonal KTN, the microscopic picture of the effect of ferroelectric domains on thermal conductivity is elaborated. Further study on TBR calculation reveals that KTN DWs possess a large ratio of the thermal switch and have great potential for application over a broad temperature range.

## 2. Experimental and Theoretical Details

### 2.1. Crystal Growth and Thermal Conductivity Measurement

KTa_0.6_Nb_0.4_O_3_ crystal is grown by the Czochralski method in this work. K_2_CO_3_, Ta_2_O_5_, and Nb_2_O_5_ with a 99.99% purity were used as raw materials. The polycrystalline powder was prepared in a special ratio and stored in a platinum crucible. The process was carried out in air, and the TDL H50AC crystal growth furnace was used for growth. First, the crucible was heated by using a medium-frequency heater with adjusted power and kept at a constant temperature for a period of time. After the crystal diameter reached a certain value, we kept the pulling rate to 0.3–0.5 mm/h and rotated the crystal at 6–8 rpm during growth. The crystal was cooled down to room temperature at a rate of 40 °C/h after the process of growth was completed [18]. The KTN single crystal was essentially a solid solution of KTaO_3_ and KNbO_3_. Therefore, uniform KTN crystals are difficult to achieve under current experimental conditions [19]. Both of the Ta/Nb ratios in the raw materials and the temperature fluctuations during the crystal growth process can lead to variations in the Ta/Nb composition ratio, and we provided the detail in our previous work [18]. After the measurement of an electric probe microanalysis and X-ray powder diffraction, we obtained tetragonal KTN crystals with an Nb concentration of 0.4.

The specific heat and thermal diffusion coefficient of KTa_0.6_Nb_0.4_O_3_ was measured by a differential scanning calorimeter (NETZSCH, Selbu, Germany) and laser flash method (NETZSCH, Selbu, Germany) over a temperature range from 50 to 500 K for wafer samples with dimensions of 10 mm × 10 mm × 2 mm and having both sides polished, was used to carry out the measurement. To reduce the reflection of the laser pulse from the crystal surface and to increase the absorption of pulse energy, the surface of the sample was uniformly sprayed with a graphite layer. The value of thermal conductivity was obtained using $\kappa =\lambda \rho C_{p}$, where λ,ρ and $C_{p}$ denote the thermal diffusion coefficient, the density, and the specific heat of KTa_0.6_Nb_0.4_O_3_, respectively.

### 2.2. Computational Method

**Figure 1:**
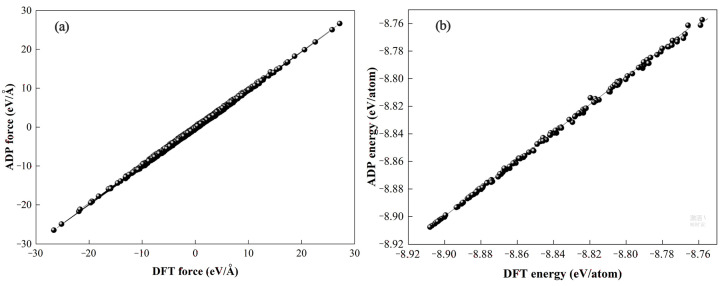
(**a**)The force comparison between DFT calculation and ADP force field for KTN. (**b**) The energy comparison between DFT calculation and ADP force field for KTN.

In order to survey the thermal conductivity of mono- and multidomain structures of KTN, we implement the NEMD calculation embedded in Large-scale Atomic/Molecular Massively Parallel Simulator software (LAMMPS, Albuquerque, NM, USA) [20]. Angular-dependent potential (ADP) was selected to describe the bond interaction in the ferroelectric perovskite oxide KTN crystal [21,22,23]. The parameter for ADP is generated with the help of the potfit code implementing force-matching method developed by Peter Brommer [24,25]. To verify the accuracy of the ADP force field, the energy and force comparison between the DFT calculation (implemented in Vienna Ab Initio Simulation Package (VASP)) [26,27] and ADP force field for KTN are provided in Figure 1, from which the considerable accuracy of the ADP force field can be witnessed. Furthermore, we have calculated and compared the lattice constants and elastic modulus as well as the Debye temperature of the KTN crystals between the ADP results and DFT results. The results are listed in Table 1 and Table 2, and good consistency was obtained indicating the validation of the ADP force field in the present work.

**Figure 2:**
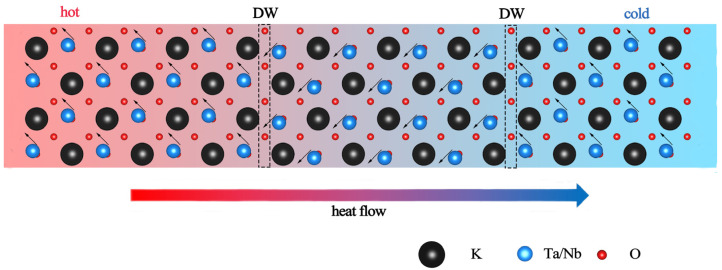
Schematic of ferroelectric arrangement containing “head-to-tail” 90° DWs. The direction of heat flow is set as perpendicular to the plane of DW.

To perform the NEMD calculation on the multidomain structure, the supercell model of KTN with 90° DWs introduced is shown in Figure 2, which indicates the (110) plane of KTN.

In a classical NEMD method, the thermal conductivity of mono- and multidomain KTN was extracted with the aid of Fourier’s law [30,31]:

(1) $$\kappa =-\frac{J}{\nabla T}$$

where J is the heat flux along the *z*-axis, and ∇T represents the temperature gradient. The heat flux J can be determined by the energy transfer rate:

(2) $$J=\frac{dE/dt}{S}$$

where *E* denotes accumulated energy, and *S* is the supercell cross-sectional area.

We used a 10 × 10 × 58 supercell system and applied fixed boundary conditions on the *z*-axis with periodic boundary conditions for the *x*- and *y*-axis. After conjugate gradient algorithm (GGA)-based energy minimization, the system was equilibrated at constant temperature (300 K) and pressure (0 Pa) for 1 ns under a Nose-Hoover barostat and thermostat (NPT) ensemble. Then, the temperatures of the cold and hot ends were set as 275 K and 325 K, respectively, via the Nose-Hoover thermostatting scheme (NVT ensemble). A microcanonical (NVE) ensemble was used for the region between the cold and hot ends. The whole simulation was performed for 2 ns to establish the steady state of the dynamic thermal transport in the system.

## 3. Results and Discussion

### 3.1. Thermal Conductivity of Tetragonal Mono- and Multi-Domain KTN

**Figure 3:**
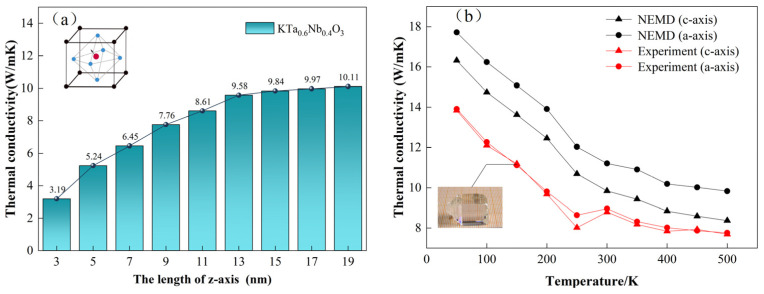
(**a**) Length-dependent thermal conductivity of monodomain ferroelectric KTN crystal with spontaneous polarization. (**b**) Temperature-dependent thermal conductivity of ferroelectric KTN crystal along *c*- and *a*-axis. Both calculated and experimental results are provided and compared.

The thermal conductivity of ferroelectric monodomain KTN was first studied. NEMD calculations were performed to study the length-dependent variation of thermal conductivity along the *c*-axis. As shown in Figure 3a, the results of thermal conductivity were increased with the length of the *c*-axis, achieving a stabilized value of 9.84 W/(m·k) with a length larger than 15 nm. Thus, the supercell configuration employed in the present work makes sure that the *c*-axis length of our simulation box was significantly longer than the phonon mean free path, which can avoid finite-size effects [32,33,34]. The comparisons between the theoretical and experimental results of thermal conductivity are provided in Figure 3b. With the temperature increased from 50 K to 500 K, the experiment value along <001> direction (*c*-axis) of KTN was decreased from 13.91 to 7.76 W/(m·k). Our calculated values decreased from 16.32 to 8.37 W/(m·k), which were well in accordance with not only the present experimental results but also a previous study by Wang et al. [35] validating the availability of the present simulation. To explore the anisotropic thermal conductivity of KTN, experiment measurements along <100> direction (*a*-axis) are also provided. The difference between the two axes was not significant due to the fact that the disordered multidomain samples in the experiment tended to demonstrate isotropic behavior. By contrast, obvious anisotropy of thermal conductivity was theoretically predicted for the tetragonal monodomain KTN, as indicated in Figure 3b.

It is worth noting that significant changes in experimental values occurred at 275 K, which corresponded to the Curie temperature that the structural symmetry of KTN transits from paraelectric cubic phase to ferroelectric tetragonal phase with decreased temperature [17,36]. Disordered domain structures were generated in the tetragonal phase of KTN due to the spontaneous polarization of the electric dipole. Thus, it was important to probe the effect of domain structure on the thermal transport behavior of KTN crystals.

**Figure 4:**
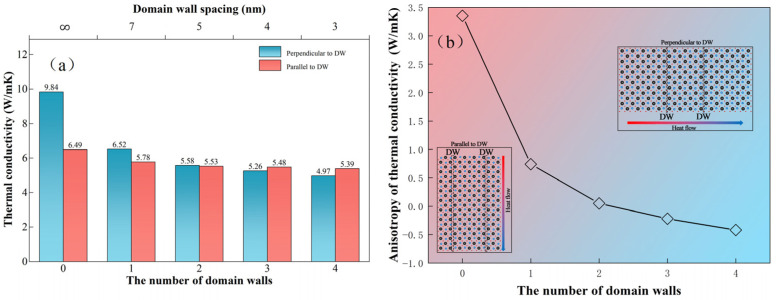
(**a**) The anisotropic thermal conductivity of multidomain KTN with the number of DWs at 300 K. The top *x*-axis indicates the variation of thermal conductivity by DWs spacing (**b**) The anisotropic thermal conductivity along with the number of DWs and the schematic diagram of the model with different heat flow directions.

In the present work, different numbers of 90° DWs were introduced to our simulation box and the NEMD method was performed to calculate thermal conductivity. The results are shown in Figure 4. Different thermal conductivity of KTN existed in the directions perpendicular and parallel to DW, and it can be deduced from Figure 4b that the anisotropic behavior was decreased with an increasing number of DWs [37,38]. The value of both directions was decreased with the number of DWs, from 9.84 W/(m·k) to 4.97 W/(m·k) for the perpendicular direction, and from 6.49 W/(m·k) to 5.39 W/(m·k) for the parallel direction, respectively. The results show that, for the perpendicular direction, the thermal conductivity decreases significantly with a single DW and was reduced inconspicuously with the increased number of DWs [13]. For the parallel direction, the value of thermal conductivity decreased insignificantly with the DWs numbers. This indicated that the DWs can be considered an efficient way to regulate the heat flow in the direction perpendicular to the DWs, thus having the pivotal role in the operation of potential thermal switch [14]. In Figure 4a, thermal conductivity depended on the DW spacing (i.e., the periodicity of DWs) is also given. With the DWs spacing increased, it indicated fewer DWs in constant length, which could deduce less thermal resistance, and thermal conductivity was thus increased accordingly.

To further understand the effect of DWs on the thermal conductivity of ferroelectric KTN crystal, the vibrational density of states (VDOS) for each type of atom is provided. The phonon VDOS was obtained using the Fourier transform of the atomic velocity autocorrelation function and calculating it according to the following equation [39].

(3) VDOS(v)=∫γ(t)exp(−2πivt)dt

where v represents the phonon frequency and γ indicates the velocity autocorrelation function, which can be calculated using the following equation [40].

(4) $$\gamma \left(t\right)=\frac{\sum_{i}v_{i}\left(0\right)\cdot v_{i}\left(t\right)}{\sum_{i}v_{i}\left(0\right)\cdot v_{i}\left(0\right)}$$

where $v_{i}\left(0\right)$ and $v_{i}\left(t\right)$ are the velocity of the *i*th atom at the initial state and time *t*, respectively.

**Figure 5:**
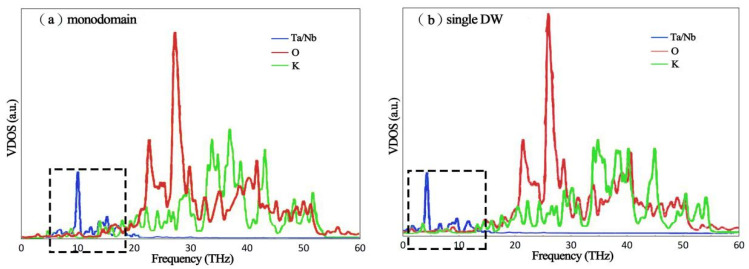
Contribution of each atom to VDOS of KTN: (**a**) monodomain (**b**) single DW.

As mentioned above, the thermal conductivity of KTN was reduced considerably by a single DW. Thus, the VDOS comparison of monodomain and single DW is worth studying. The results are shown in Figure 5. For the homogeneous configuration (DW-free) of KTN, the vibrational distributions of O and K atoms dominate the mid- and high-frequency range (20–60 THz), while the contribution of the B-site atom (Ta/Nb) was mainly located at the low-frequency range (around 10 THz). For ferroelectric KTN with a single DW, the frequency distributions of O and K atoms were similar to the monodomain state, while the vibration of B-site atom (Ta/Nb) shifts to a lower frequency range (around 5 THz), inducing the decreased VDOS overlap of Ta/Nb and oxygen atoms. Since the VDOS overlap between Ta/Nb and oxygen atoms was considered to determine the number of elastic phonon channels, therefore, the thermal conductivity of a single DW was decreased with decreased phonon transport channels [41,42].

### 3.2. Study on Thermal Boundary Resistance and Thermal Switch of DW

The DW between adjacent domains can act as a scattering plane for the incident phonons, which created TBR that can be switched on or off by an external electric field [14]. For 90° DW of ferroelectric KTN, the TBR property should be studied in order to explore the potential application of thermal switches.

**Figure 6:**
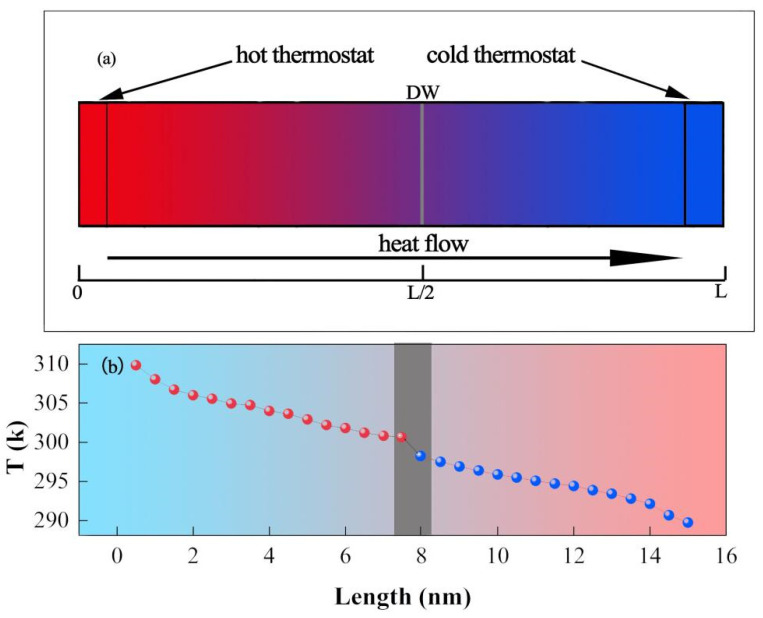
(**a**) Schematic view of KTN supercell with single 90° DW for TBR calculation. (**b**) Temperature gradient along the *z*-axis for single 90° DW of KTN.

It is schematically shown in Figure 6a that the heat source is fixed at the left end so that it produced a steady state heat flow along the *c*-axis and we removed the heat flow by fixing the cold thermostat at the right end of the *c*-axis, with *L* denoting the length along the direction of heat flow transport. Single DWs were located at *L*/2 to prevent the results from being influenced by the temperature of the hot and cold ends. Performing the NEMD calculation, the results of the temperature profile in Figure 6b show that the linear relation can be observed far away from the DW region. An abrupt change of temperature gradient was witnessed around the DW, which accorded with previous studies of TBR [43,44].

Based on the common formulation of Kapitza resistance [45,46], we calculated the TBR of ferroelectric DW. At the interface of DW, the thermal boundary resistance is expressed as

(5) $$R=\frac{dT}{J}$$

where the heat flux entering (exiting) the interface is represented by *J* and the temperature gradients near the interface are denoted by *dT*.

All quantities that appeared in the above equations can be obtained by NEMD simulation. The TBR value of present ferroelectric DW (*R*_DW_) is 2.29 × 10^−9^ K·m^2^/W, which is significantly larger than the theoretical results of 2.9 × 10^−10^ K·m^2^/W of PbTiO_3_ [14] and comparable to the experimental results of 5.0 × 10^−9^ K·m^2^/W of PbTiO_3_ [15]. By contrast, the thermal resistance of monodomain KTN (*R*_0_) is calculated as 0.48 × 10^−9^ K·m^2^/W.

Analogous to binary code in an electronic circuit, phononic binary code “0” or “1” can be achieved by writing or erasing DWs [14], corresponding to the low and high conductive states. The performance of the phonon thermal switch is characterized by the ratio between the two states, which can be obtained as:

(6) $$\frac{R_{\mathrm{high}}}{R_{\mathrm{low}}}=1+\frac{R_{\mathrm{DW}}}{L/k_{\mathrm{KTN}}}$$

We took the value *L* = 6 nm (half of the simulation length without the interface thickness) and finally obtained the thermal switch ratio of *R*_high_/*R*_low_ = 4.76.

Compared to the DWs’ TBR study of PbTiO_3_ [13], the present ratio of thermal switch in KTN is about fourfold that of PbTiO_3_, implying the outstanding performance of regulating the heat flow by ferroelectric DWs of KTN. It is worth mentioning that the low Curie temperature of PbTiO_3_ and most ferroelectric materials largely limits their application, while the Curie temperature of solid-solution KTN crystal can be dynamically controlled by regulating the ratio of Ta/Nb. Thus, a solid-state thermal switch over a broad temperature (including room temperature) can be obtained based on ferroelectric KTN crystals.

## 4. Conclusions

In the present work, we investigated the thermal transport behavior and thermal boundary resistance of ferroelectric DWs in tetragonal KTN single crystal, both experimentally and theoretically. By performing a NEMD simulation on the mono- and multidomain structure of KTN, our results on thermal conductivity are consistent with the experimental measurement of KTa_0.6_Nb_0.4_O_3_, ferroelectric phase of KTN being obtained by the Czochralski method. The value of the multidomain structure was decreased, obviously, by a single DW and decreased inconspicuously with the increased number of DWs. The VDOS results show that the frequency of the Ta/Nb atom shifting to the lower side inhibits the energy exchange, leading to lower thermal conductivity. To explore the performance of the thermal switch, the calculation of interfacial thermal resistance was carried out. The presence of a single DW increased the thermal resistance by fourfold, which produces a large thermal switch ratio of 4.76 based on ferroelectric KTN crystals. On the basis of the present study, we anticipate that the goal of dynamically tuning thermal flux and phonon scattering behavior over a broad temperature range can be achieved through electrically actuated ferroelectric DWs in KTN crystal.

## Figures and Tables

**Table 1:** Comparison of lattice constant between ADP results and previous DFT results.

	Experimental Values	DFT Results [28]	ADP Results
a=b(Å)	4.03	4.03	4.06
c(Å)	4.23	4.11	4.18
$V\left({Å}^{3}\right)$	68.47	66.72	68.56

**Table 2:** Comparison of elastic stiffness (constant *c*_ij_ in GPa) and Debye temperature *θ*_D_ (in K) between ADP results and previous DFT results.

	DFT Results [28]	ADP Results
*c*_11_	424.83	425.27
*c*_33_	121.38	123.11
*c*_44_	82.86	84.57
*c*_12_	68.05	69.86
*c*_13_	80.70	83.59
*c*_66_	93.61	95.49
*θ*_D_	654.34 [29]	671.68

## Data Availability

Not applicable.

## References

[1] Merz W.J. (1954). Domain formation and domain wall motions in ferroelectric BaTiO_3_ single crystals. Phys. Rev..

[2] Vassighi A., Sachdev M. (2006). Thermal runaway in integrated circuits. IEEE Trans. Device Mater. Reliab..

[3] Li N., Ren J., Wang L., Zhang G., Hänggi P., Li B. (2012). Colloquium: Phononics: Manipulating heat flow with electronic analogs and beyond. Rev. Mod. Phys..

[4] Toberer E.S., Baranowski L.L., Dames C. (2012). Advances in thermal conductivity. Annu. Rev. Mater. Res..

[5] Luckyanova M.N., Garg J., Esfarjani K., Jandl A., Bulsara M.T., Schmidt A.J., Minnich A.J., Chen S., MSDresselhaus M.S., Chen G. (2012). Coherent phonon heat conduction in superlattices. Science.

[6] Maldovan M.J. (2015). Phonon wave interference and thermal bandgap materials. Nat. Mater..

[7] Lee S., Hippalgaonkar K., Yang F., Hong J., Ko C., Suh J., Liu K., Wang K., Urban J.J., Zhang X. (2017). Anomalously low electronic thermal conductivity in metallic vanadium dioxide. Science.

[8] Shrestha R., Luan Y., Luo X., Shin S., Zhang T., Smith P., Gong W., Bockstaller M., Luo T., Chen R. (2020). Dual-mode solid-state thermal rectification. Nat. Commun..

[9] Shrestha R., Luan Y., Shin S., Zhang T., Luo X., Lundh J.S., Gong W., Bockstaller M.R., Choi S., Luo T. (2019). High-contrast and reversible polymer thermal regulator by structural phase transition. Sci. Adv..

[10] Mante A.J.H., Volger J. (1971). Phonon transport in barium titanate. Physica.

[11] Weilert M., Msall M., Wolfe J.P., Anderson A.C. (1993). Mode dependent scattering of phonons by domain walls in ferroelectric KDP. Z. Phys. B Condens. Matter.

[12] Weilert M., Msall M., Anderson A.C., Wolfe J.P. (1993). Phonon scattering from ferroelectric domain walls: Phonon imaging in KDP. Phys. Rev. Lett..

[13] Royo M., Escorihuela-Sayalero C., Íñiguez J., Rurali R.J. (2017). Ferroelectric domain wall phonon polarizer. Phys. Rev. Mater..

[14] Seijas-Bellido J.A., Escorihuela-Sayalero C., Royo M., Ljungberg M.P., Wojdeł J.C., Íñiguez J., Rurali R.J. (2017). A phononic switch based on ferroelectric domain walls. Phys. Rev. B.

[15] Langenberg E., Saha D., Holtz M.E., Wang J.J., Bugallo D., Ferreiro-Vila E., Paik H., Hanke I., Ganschow S., Muller D.A. (2019). Ferroelectric domain walls in PbTiO_3_ are effective regulators of heat flow at room temperature. Nano Lett..

[16] Hopkins P.E., Adamo C., Ye L., Huey B.D., Lee S.R. (2013). Effects of coherent ferroelastic domain walls on the thermal conductivity and Kapitza conductance in bismuth ferrite. Appl. Phys. Lett..

[17] Wang X., Wang J., Yu Y., Zhang H., Boughton R.I. (2006). Growth of cubic KTa_1−x_Nb_x_O_3_ crystal by Czochralski method. J. Cryst. Growth.

[18] Wang J., Guan Q., Wei J., Wang M., Liu Y.J. (1992). Growth and characterization of cubic KTa_1-x_Nb_x_O_3_ crystals. J. Cryst. Growth.

[19] Nosé S. (1984). A unified formulation of the constant temperature molecular dynamics methods. J. Chem. Phys..

[20] Evans D.J. (1982). Homogeneous NEMD algorithm for thermal conductivity—Application of non-canonical linear response theory. Phys. Lett. A.

[21] Mishin Y., Lozovoi A.J. (2006). Angular-dependent interatomic potential for tantalum. Acta Mater..

[22] Pokatashkin P., Kuksin A., Yanilkin A. (2015). Angular dependent potential for α-boron and large-scale molecular dynamics simulations. Model. Simul. Mater. Sci. Eng..

[23] Apostol F., Mishin Y. (2010). Angular-dependent interatomic potential for the aluminum-hydrogen system. Phys. Rev. B.

[24] Brommer P., Gähler F. (2007). Potfit: Effective potentials from ab initio data. Model. Simul. Mater. Sci. Eng..

[25] Brommer P., Gähler F. (2006). Effective potentials for quasicrystals from ab-initio data. Philos. Mag..

[26] Kresse G., Furthmüller J. (1996). Efficient iterative schemes for ab initio total-energy calculations using a plane-wave basis set. Phys. Rev. B.

[27] Kresse G., Furthmüller J. (1996). Efficiency of ab-initio total energy calculations for metals and semiconductors using a plane-wave basis set. Comp. Mater. Sci..

[28] Wang Y., Shen Y., Zhou Z. (2011). Structures and elastic properties of paraelectric and ferroelectric KTa_0. 5_Nb_0.5_O_3_ from first-principles calculation. Phys. B.

[29] Yang W., Han J., Wang L., Yangqing S., Linjun L., Yuqiang Y., Haidong L., Liangyu C. (2017). Effect of ordered B-site cations on the structure, elastic and thermodynamic properties of KTa_0. 5_Nb_0. 5_O_3_ crystal. Appl. Phys. A.

[30] Ikeshoji T., Hafskjold B. (1994). Non-equilibrium molecular dynamics calculation of heat conduction in liquid and through liquid-gas interface. Mol. Phys..

[31] Jund P., Jullien R.J. (1999). Molecular-dynamics calculation of the thermal conductivity of vitreous silica. Phys. Rev. B.

[32] Wojdeł J.C., Iniguez J.J. (2014). Ferroelectric transitions at ferroelectric domain walls found from first principles. Phys. Rev. Lett..

[33] Schelling P.K., Phillpot S.R., Keblinski P.J. (2002). Comparison of atomic-level simulation methods for computing thermal conductivity. Phys. Rev. B.

[34] Sellan D.P., Landry E.S., Turney J., McGaughey A.J., Amon C.H. (2010). Size effects in molecular dynamics thermal conductivity predictions. Phys. Rev. B.

[35] Wang X.P., Wang J.Y., Zhang H.J., Yu Y.G., Wu J., Gao W.L., Boughton R.I. (2008). Thermal properties of cubic KTa_1−x_Nb_x_O_3_ crystals. J. Appl. Phys..

[36] Wang M., Yang Z.H., Wang J.Y., Liu Y.G., Guan Q.C., Wei J.Q. (1992). The thermal properties of KTa_1-x_Nb_x_O_3_ crystal. Ferroelectrics.

[37] Fonseca L.R.C., Liu D., Robertson J. (2008). p-type Fermi level pinning at a Si: Al_2_O_3_ model interface. Appl. Phys. Lett..

[38] Seijas-Bellido J.A., Íñiguez J., Rurali R. (2019). Anisotropy-driven thermal conductivity switching and thermal hysteresis in a ferroelectric. Appl. Phys. Lett..

[39] Dickey J.M., Paskin A. (1969). Computer simulation of the lattice dynamics of solids. Phys. Rev..

[40] Haile J.M. (1993). Molecular Dynamics Simulation: Elementary Methods.

[41] Kim W. (2015). Strategies for engineering phonon transport in thermoelectrics. J. Mater. Chem. C.

[42] Pennec Y., Rouhani B.D., Larabi H., Akjouj A., Gillet J., Vasseur J., Thabet G.J. (2009). Phonon transport and waveguiding in a phononic crystal made up of cylindrical dots on a thin homogeneous plate. Phys. Rev. B.

[43] Rurali R., Colombo L., Cartoixà X., Wilhelmsen Ø., Trinh T.T., Bedeaux D., Kjelstrup S. (2016). Heat transport through a solid–solid junction: The interface as an autonomous thermodynamic system. Phys. Chem. Chem. Phys..

[44] Dettori R., Melis C., Cartoixà X., Rurali R., Colombo L. (2016). Thermal boundary resistance in semiconductors by non-equilibrium thermodynamics. Adv. Phys-X..

[45] Pollack G.L. (1969). Kapitza resistance. Rev. Mod. Phys..

[46] Nan C.W., Birringer R. (1998). Determining the Kapitza resistance and the thermal conductivity of polycrystals: A simple model. Phys. Rev. B.

